# Long-Term Results of Ultrasound-Guided Radiofrequency Ablation of Benign Thyroid Nodules: State of the Art and Future Perspectives—A Systematic Review

**DOI:** 10.3389/fendo.2021.622996

**Published:** 2021-05-26

**Authors:** Hervé Monpeyssen, Ahmad Alamri, Adrien Ben Hamou

**Affiliations:** ^1^ American Hospital of Paris, Thyroid Unit, Neuilly-sur-Seine, France; ^2^ Department of Endocrinology, Diabetology and Nutrition, Paris Saint-Joseph Hospital, Paris, France; ^3^ Thyroid and Endocrine Tumors Department, Institute of Endocrinology, Pitié-Salpêtrière Hospital, Paris, France

**Keywords:** benign thyroid nodule, non-functioning thyroid nodule, minimally invasive treatment, thermal ablation, ultrasound-guided radiofrequency ablation

## Abstract

**Background:**

Nearly 20 years after the first feasibility study, minimally invasive ultrasound (US)-guided therapeutic techniques are now considered as a safe and effective alternative to surgery for symptomatic benign thyroid nodules. Radiofrequency ablation (RFA) is one of the most widely used treatment in specialized thyroid centers but, due to the relatively recent introduction into clinical practice, there are limited long-term follow-up studies. Aim of our work was to review the outcomes of RFA on solid nonfunctioning and on autonomous thyroid nodules (AFTN) on a long-time period for assessing the results in term of efficacy, complications, and costs and to compare them to the current indications of RFA.

**Methods:**

A systematic review was performed using EMBASE and Medline library data between 2008 and 2021. Seventeen studies evaluated RFA for the treatment of benign solid (nonfunctioning or autonomous) thyroid nodules, with an at least 18 months of follow-up. Data extraction and quality assessment were performed by two endocrinologist according to PRISMA guidelines. Anthropometric data, safety and efficacy parameters were collected.

**Results:**

The majority of the studies was retrospective study and reported 933 nodules, mostly solid. Baseline volume ranged between 6.1 ± 9.6 and 36.3 ± 59.8 ml. Local analgesia was used and the time duration of the treatment was between 5 ± 2 and 22.1 ± 10.9 min. The volume reduction rate at 12 months ranged from 67% to 75% for the nodule treated with a single procedure and reached to 93.6 ± 9.7% for nodules treated with repeat ablations. The regrowth rate at 12 months ranged from 0% to 34%.

**Conclusion:**

All the studies under examination consistently validated the long-term clinical efficacy and the substantial safety of RFA for the treatment of benign thyroid nodules. Thermal ablation, however, is an operator-dependent technique and should be performed in centers with specific expertise. The selection of the patients should be rigorous because the nodule size and the structural and functional characteristics influence the appropriateness and the outcomes of the treatment. Future perspectives as the treatment of micro-papillary thyroid cancer or cervical recurrence need further investigations.

## Introduction

Thyroid nodules (TN) are palpable in 4% to 7% of the general population and are identifiable by ultrasound (US) in 67% of cases (1, 2). If thyroid fine-needle aspiration (FNA) provides the cytological diagnosis of a benign thyroid nodule (Bethesda Category II), various therapeutic options are now available for symptomatic patients. The American Thyroid Association suggests surgical lobectomy if the long-axis of the nodule is >4 cm while European Guideline recommendations (3) consider as an alternative therapeutic option the treatment of symptomatic nodules with US-guided thermal ablation (TA) techniques (4). During the last years, a mounting evidence demonstrated the efficacy of radiofrequency ablation (RFA) (5), laser ablation (LTA) (6), high-intensity focused ultrasound [(HIFU) (7), and microwave (MWA) (8)]. This increasing interest in nonsurgical procedures is due to the present excess of thyroid surgery for benign lesions. Surgical management, indeed, is expensive, may be complicated by dysphonia and hypocalcemia (9, 10), and is followed in the majority of cases by need of lifelong replacement therapy (11, 12).

Nearly 20 years after the first publications, mini invasive ultrasound (US)-guided techniques are now considered as a safe and effective alternative treatment to surgery for symptomatic benign nodules. RFA is one of the most widely used procedures in specialized centers (13–16) as may be applied to all types of nodules (solid or partially fluid, autonomous or “cold”). The results in term of volume reduction rate (VRR) are satisfactory (>50% of nodule volume reduction) and are paralleled by the improvement of pressure symptoms and cosmetic concerns. In most of the available prospective and retrospective studies follow-up (FU) is up to 12 months, a time period insufficient to prove the real effectiveness of the technique in preventing late nodule regrowth. On the basis of a robust evidence (17–19), TA is now proposed as a possible first-line treatment option for selected patients by the Korean Health Authorities (20), the American Association of Clinical Endocrinologists, the American College of Endocrinology, the Italian Association of Clinical Endocrinologists (AME) (21), and, more recently, by the European Thyroid Association (ETA) (3).

The aim of our work was to review the therapeutic effects of RFA on solid nonfunctioning or autonomously functioning (AFTN) thyroid nodules on a long-time period setting the minimum FU at 18 months. A comprehensive literature search was conducted by consulting the PubMed, EMBASE, and the Cochrane Library data bases on original articles published in English concerning the effects of RFA on nonfunctioning thyroid nodules and AFTN between 2008 and 2021. Full texts were reviewed to exclude articles concerning systematic review and case reports. A dedicated systematic review of the literature assessed the frequency of nodule regrowth after RFA in the long term.

## Methods

### Literature Search

**Figure 1:**
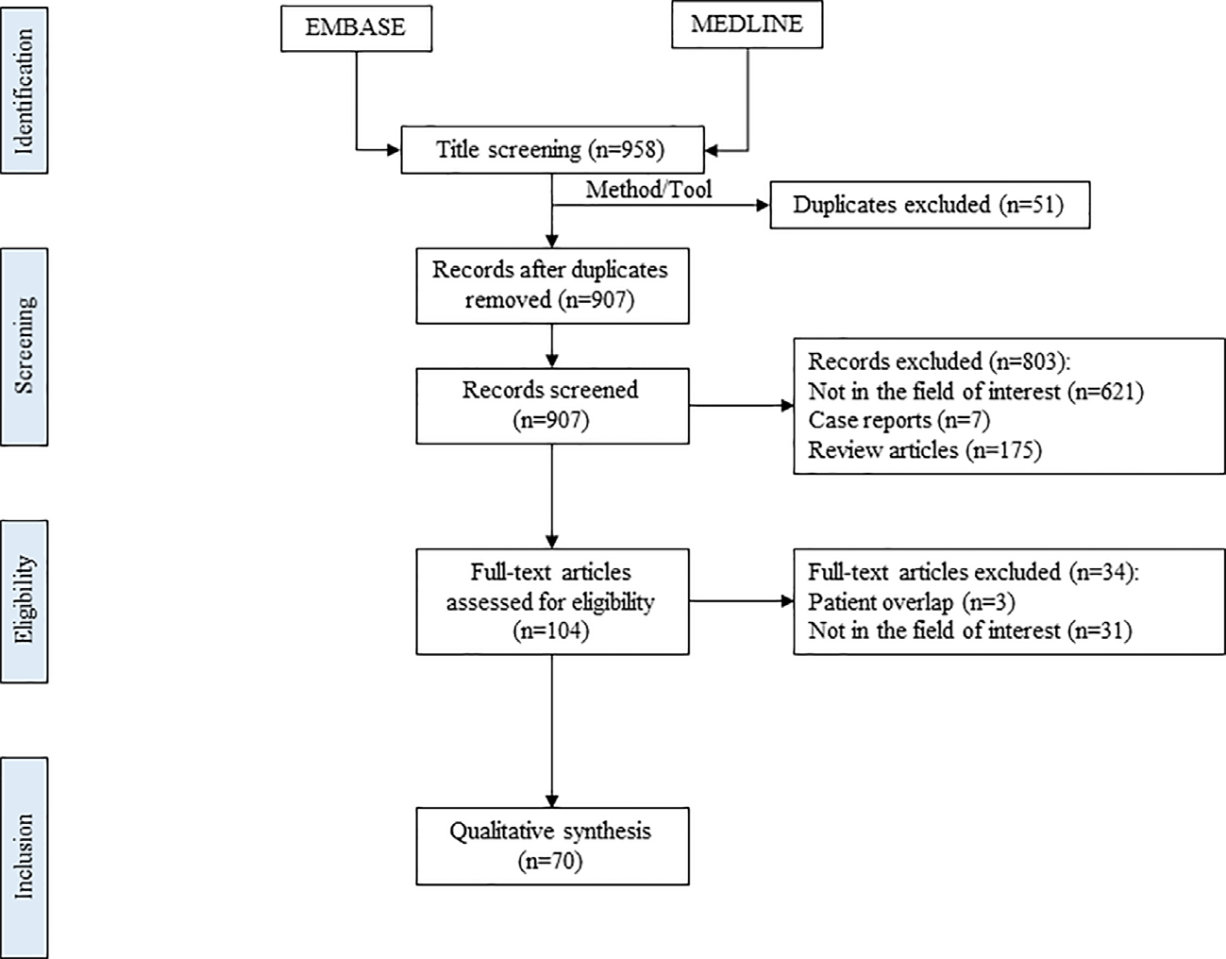
Flowchart of the study selection process.

By consulting the PubMed, EMBASE, and the Cochrane databases, we selected articles in the period between 2008 and 2021. We searched by the following algorithm “thyroid nodule’/exp AND (‘radiofrequency thermal ablation’/exp OR ‘radiofrequency thermal ablation’ OR rfa OR (‘radiofrequency’/exp AND (‘thermal ablation’/exp OR (thermal AND ablation)))) NOT (‘thyroid nodule’/exp AND (‘radiofrequency thermal ablation’/exp OR ‘radiofrequency thermal ablation’ OR rfa OR (‘radiofrequency’/exp AND (‘thermal ablation’/exp OR (thermal AND ablation)))) AND (‘case report’/de OR ‘meta-analysis’/de)) AND [embase]/lim NOT ([embase]/lim AND [medline]/lim)” (**Figure 1**). Two endocrinologists, ABH and HM, with 5 and 31 years of experience in thyroid imaging, respectively, independently performed the literature search and selection.

### Inclusion Criteria

Articles fulfilling the following criteria were included: (1) patients with a benign thyroid nodule that has not been treated with a different method TA, surgery, RIA, or PEI and, (2) FU data for more than 18 months after RFA.

### Exclusion Criteria

The following criteria were excluded (**Table 1**): (1) age <15 years or >80 years; (2) women who were pregnant or lactating; (3) patients who were incapable to maintain a stable position while the neck is in hyperextension; (4) history of irradiation on the neck; (5) preexisting vocal cord palsy; (6) tattoos, skin moles, or scars on the neck area; (7) suspicion of malignancy (US or cytological); (8) if macro calcification or fluid collection are present; (9) if the lesion is in contact with the trachea, the esophagus or the carotid artery; (10) case reports or case series including fewer than 20 patients; (11) articles written in a language other than English; (12) articles lacking reference standards based on cytological test; (13) articles with overlapping populations; (14) letters, editorials, conference abstracts, systematic reviews or meta-analyses, consensus statements, guidelines, and review articles.

**Table 1:** Inclusion and exclusion criteria in patients with thyroid nodules before RFA procedure.

Inclusion	Exclusion
≥18 years old	<18 years old
Written inform consent	Pregnancy
Functional and/or compressive complaints:	Nodule classified as Bethesda I, III, IV, V, VI
Anterior cervical discomfort	
Dyspnea	
Dysphagia	
Dysphonia	
Cosmetic complaint	Ultrasound (US) aspect compatible with medullary thyroid cancer and positive calcitonin
Two benign cytology or biopsy (Bethesda II)	Nodule too close to the “danger zone”
Autonomous thyroid nodule	Not available for thermal ablation after US evaluation
Refusal/contraindication for surgery	Multinodular thyroid
Not available for radioiodine treatment	
Not available for percutaneous ethanol injection (PEI)	
History of neck surgery or radiotherapy	

### Patient Assessment Before RFA Procedure

Nodules were classified according to the Thyroid Image Reporting and Data System (TI-RADS) classification, and the vascularization of the nodules were assessed by color flow Doppler, with the vascularization classified using a four-grade scale (grade 0: no vascularity; grade 1: perinodular vascularity only; grade 2: intranodular vascularity <50%; and grade 3: intranodular vascularity >50%). Two fine needle aspiration cytology (FNA) guided by US were performed with a minimum interval of 90 days, according to the described technique. Before the procedure, US imaging was performed by high frequency linear US transducer. All of the nodules treated were predominantly or even completely solid (≤10% of fluid component) (22). Five series included autonomous or even toxic nodules (4.1% to 100% of the treated nodules). Some series report several nodules treated in the same session. The mean volume of the nodules was between 6 and 37 ml. The biological tests performed before the procedure were: complete blood count, blood calcium level (mg/L), calcitonin (pg/ml), TSH (µIU/ml), fT3 (pg/ml), fT4 (ng/dl), anti-TPO antibodies (IU/L). Patients that had a TSH level in the low normal range were tested by I^123^ nuclear imaging to eliminate the presence of hyper functioning nodules. A clinical examination was performed to assess pressure symptoms or cosmetic damage (on visual analogic scale); pressure symptoms were defined as a persisting dysphagia or cervical constriction, while cosmetic damage was defined as the by physicians using a four-grade score (1. no palpable nodule; 2. palpable nodule without cosmetic damage; 3. the presence of a cosmetic problem on swallowing; 4. easily detected cosmetic problem).

**Figure 2:**
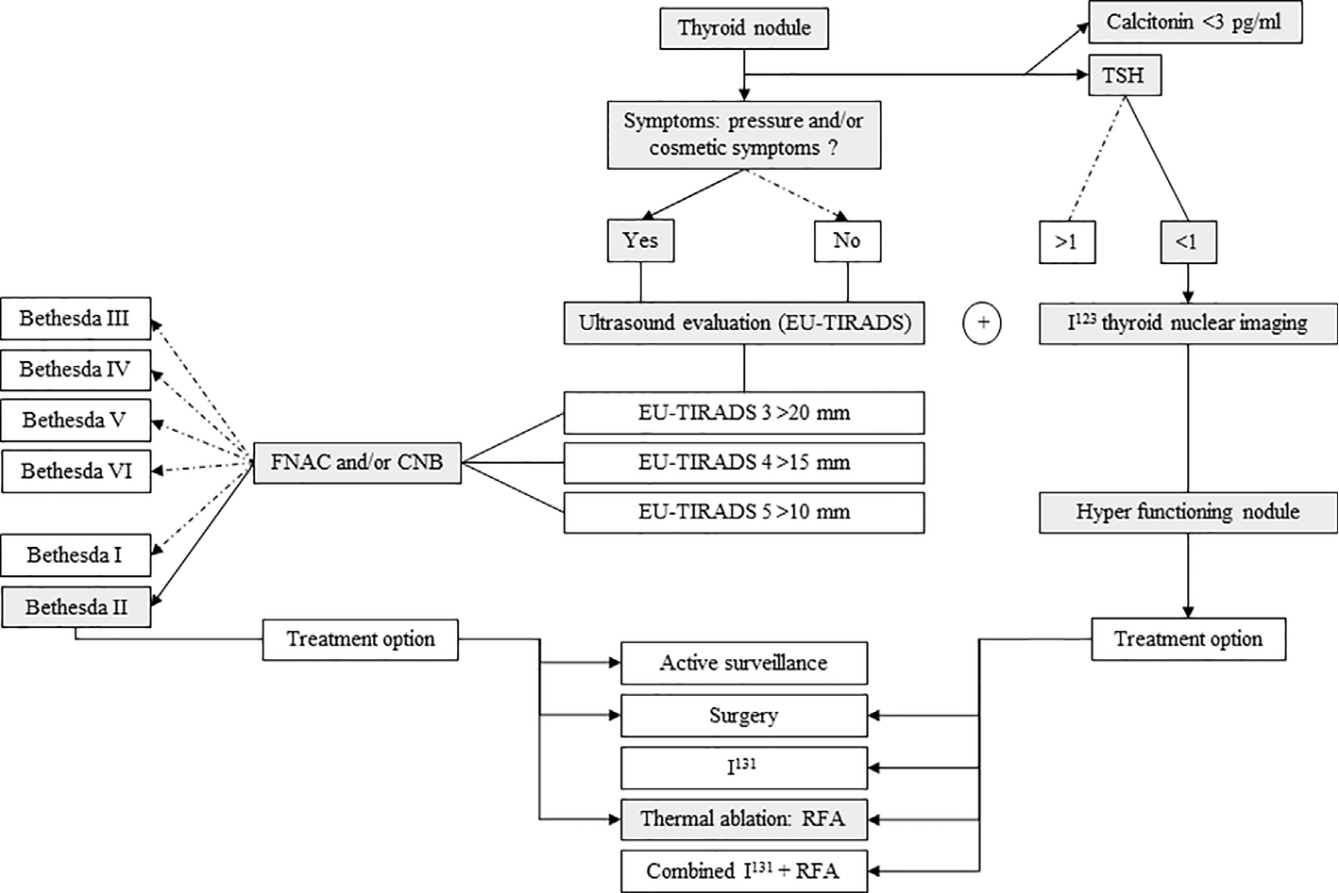
Algorithm for the treatment of symptomatic benign thyroid nodules, based on the 2020 European Thyroid Association (ETA) guidelines. FNAC, fine-needle aspiration cytology; CNB, core needle biopsy; RFA, radiofrequency ablation. TSH expressed as µUI/ml.

We proposed an algorithm for the treatment of solid benign symptomatic thyroid nodules, based on the 2020 ETA guidelines (**Figure 2**).

### The Procedure

All procedures were outpatient, with the use of US guidance. Patients were placed in supine position, while the extension of the neck was determined by the nodule localization. The electrodes used were monopolar, except in the study by Guang et al. (22) (bipolar) and in one of the older publications, Spieza used a HOOK (umbrella-like) electrodes (23). For TA, we used different generator and an electrode cooled by a 0.9% sodium chloride solution bag at 4°. The electrode was inserted using a trans-isthmic approach starting from the deepest part of the nodule, and a moving-shot TA technique was performed (successive treatment of small parts of the nodule). In two-thirds of the studies, only local anesthesia was used. Four series reported using conscious sedation, while some studies specified the use of Midazolam. The number of procedures was very variable, Sim (24), Dobrinja (25) and Valcavi (26) performed a single procedure. Lim et al. (16) has treated some of his patients seven times. In Ben Hamou’s publication (27), one nodule received two treatments. The same is observe for Valcavi: the first procedure was stopped prematurely due to a vasovagal syncope. The power delivered during the procedure varies between 20 and 120 Watts. In the series treated as bipolar, the power used is much lower. The processing time is reported by nine teams. The duration varies between 8 and 22 minutes. In almost half of the studies, the energy delivered per milliliters of tissue treated is not available. The others use the energy delivered by nodule. Complications were evaluated during and immediately after the procedure (symptoms or clinical signs). Patients were observed for 2 to 6 h after the procedure.

## Results

### Eligibility

The initial systematic search showed 958 articles. Fifty-seven duplicates were then removed, and following that the remaining 907 articles were screened (by looking through titles and abstracts), and 104 potentially eligible articles were found. The 19 publications were then assessed for eligibility: 17 publications in which the post-treatment FU was equal to or more than 18 months. Out of the seventeen publications, four of them were prospective studies. We ruled out studies where another therapy method (surgery, RIA, percutaneous ethanol injection (PEI) or other TA techniques) had been used or had preceded RFA. At the end of the assessment 7 out of the 19 articles were excluded (1 case report/series, 2 articles with overlapping data and 4 articles with no data more than 3-year follow up). Almost half of the studies come from the same team (Asan Medical Center, Seoul). The data extracted from the selected articles are shown in **Table 2**.

**Table 2:** Characteristics of the included studies.

Authors (year)	Patients (nodules)	Mean age (years)	M/F	Type	Structure	AFTN	Mean nodule volume (mL)	E-Type	Sedation	Power (watts)	Delivered energy (kJ)	Number of procedures	Time (minutes)	VRR	FU period (months)	Percentage of Regrowth	Re-treatment
Jeong et al. Eur Radiol (2008) 18:1244–1250 (28).	236 (302)	40.9	25/211	2	S: 54.3% C: 16.2% M: 29.5%	0	6.13±9.59	1	No	20 to 70	N/A	1 to 6	5 to 30	84.1±14.93	1 to 41	N/A	x2: 20.8% x3: 6.6% >3: 2.3%
Spiezia et al. Thyroid (2009) 19:219–225 (23).	94	72.5±0.5	39/55	1	S>70%	100%	24.5±2.1	1 (Hook)	No	N/A	N/A	1 to 3	5±2	79.4±2.5	24	34%	24%
Lim et al. Eur Radiol (2013) 23:1044–1049 (16).	111 (126)	37.9±10.6	10/101	1	S>65%	N/A	9.8±8.5	1	No	30 to 120	2.9±1.99	2.2 (1 to 7)	N/A	93.6±9.7	49.4±13.6	5.6%	x2: 23.8% x3: 19% >3: 13.5%
Sung et al. Thyroid (2015) 25:112–117 (29).	44 (44)	43±14.7	2/42	2	S: 59.1% C: 36.4%	100%	18.5±30.1	1	No	15 to 70	6.41±4.31	1.8±0.9	12±5.9	81.7±13.6	19.9	N/A	x2: 27% x3: 7% >3: 1%
Dobrinja et al. Int J Endocrinol (2015) 2015:576576 (25).	64	60.47±1.89	17/47	2	S: 85.9%	0	13.81±1.86	1	Yes	60 to 80	N/A	1	98.5±6.5	67±?	24	1.5%	0
Hong et al. J Vasc Interv Radiol (2015) 26:55–61 (30).	18(36)	49.9	2/16	2	N/A	0	24.4±32.2	1	N/A	N/A	N/A	>1	N/A	70.3±16.2	18.1±12.8	N/A	N/A
Valcavi et al. Endocr Pract (2015) 21(8):887-96 (26).	40 (40)	54.9±14.3	5/35	2	S>80%	0	30±18.2	1	Yes	37.4±8.8	37.15±18.1/nodule	1 or 2	22.1±10.9	80.1±16.1	24	0	0
Sim et al. Int J Hyperthermia (2017) 33:905–910 (24).	52 (54)	44.1±13.2	5/49	2	S: 70.4% M: 20.4% C: 9.3%	0	14±12.7	1	No	N/A	N/A	1	N/A	69.6	39.4	24.1%	0
Cervelli et al. J Vasc Interv Radiol (2017) 28:1400–1408 (5).	46 (51)	56.4±11.3	15/31	1	S>75%	0	9.1±42	1	Yes	30 to 50	35.2±14.5	1 to 2	N/A	84.3 to 86.4	18	N/A	x2: 13%
Jung et al. Korean J Radiol (2018) 19:167–174 (13).	345 (345)	46±12.7	43/302	1	S: 89.9% C: 10.1%	0	14.2±13.2	1	No	78.8± 41.6	4.16±2.9	1.3±0.4	9.52±5.50	80.3±13.7	12	5.6%	x2: 20.3%
Guang et al. BMC Cancer (2019) 19:147 (22).	103 (194)	47.6±11.3	36/67	2	S	0	21.2±19.7	2	No	3 to 8	N/A	1 to 3	N/A	72.3 to 98.7	16.3±5.6	0	x2: 44.9% x3: 4.6%
Ben Hamou et al. Int J Hyperthermia (2019) 36:666–676 (27).	99 (108)	49.7±12.2	19/80	2	S: 64.8% C: 13.9% M: 21.3%	13.8%	20.4±18.6	1	Yes	35 to 55	7.8±5.8	1 to 2	18.7±10.6	75 (64.6 – 83.3)	18	4.6%	x2: 1/108 3 surgeries
Ha et al. Endocrinol Metab (Seoul) (2019) 34:169–178 (31).	16 (16)	43.8±12.3	1/15	2	S: 81.3%	0	34.6±28.5	1	No	76	3.23±4.91	1.6±0.9	8.2±17.7	y2 71.5±5.8 y5 71.8±6.9	69.6 (38 to 98)	N/A	x2: 43% x3: 12.5% >3: 6%
Hong MJ et al. J Vasc Interv Radiol (2019) 30:900–906 (32).	14	15.7±2.3	4/10	2	N/A	0	14.6±13.3	1	No	47.1±22.9	3.15±2.06	2.1±1.2	7.3±2.75	92.1±11.4	36.9±21.7	N/A	x2: 28.5% x3: 28.5% x5: 7.1%
Deandrea et al. J Clin Endocrinol Metab (2019) 104:3751–3756 (33).	215 (215)	66	33/182	2	S>70%	0	20.9 (15 –33)	1	N/A	55	55 (50 – 65)	1-2	14 (12–19)	69.8	35 (24 – 60)	4.1%	9 RFA N°2 or surgery
Aldéa Martinez et al. J Vasc Interv Radiol (2019) 30:1567–1573 (34).	24	50.17±13.6	4/20	1	S: 54.2% M: 37.5% C: 8.3%	4.1%	36.3±59.8	1	Yes	45.8±8.3	N/A	3.5±0.93	15.6±6.5	76.84±15.92	36	N/A	N/A
Bernardi et al. Thyroid (2020) 30(12):1759-1770 (17).	216	57 (17-87)	102/304	2	S : 75% M:24% C: 1%	17.1%	17.2 (.4-179)	1	N/A	N/A	1.397j/ml 0.75-24	1.12	N/A	77.1	60	20%	26 (12%) 15 surgery 15 MITT

M/F, ratio male and female; Type, 1 prospective, 2 retrospective; Structure: C, cystic nodule; S, solid nodule; M, mixed; AFTN, autonomous functioning thyroid nodule; Type of electrode: 1, monopolar; 2, bipolar; volume, initial volume of the treated nodule (mean ± SD, or median (IQR), ml); VVR, volume reduction rate in percentage (mean ± SD or median (IQR), %); FU, follow-up (months); MITT, minimally invasive treatment of thyroid; N/A, not applicable.

The following data were extracted using standardized forms according to the PRISMA guidelines (35).

The primary outcome of the current systematic review was the serial VRRs of ablated nodules over 18 months or more. A secondary outcome was a description of the adverse effects of ablation, including the rates of complications and surgery during FU after ablation.

### The Patients

The total number of patients in the cohorts included between 16 and 345 patients. One thousand seven-hundred and thirty-seven patients had a FU for more than 18 months for certain studies, some of which were multi-centric (6 studies) for a total of 1943 nodules treated. The majority of the population in all cohorts were females, the mean age ranged between 41 and 72.5 years, with two studies with patients in the seventh decade, and another in the eighth decade (72.5 years) (23) and one in children/adolescent (32).

All of the nodules treated were predominantly or even completely solid (≤10% of fluid component) (22). Five series included autonomous or even toxic nodules (4.1% to 100% of the treated nodules). Some series report several nodules treated in the same session. The mean volume of the nodules was between 6 and 37 ml.

### Endpoints of Efficacy

The Volume Reduction Ratio (VRR) is used in all series. It is calculated with the formula: initial volume (ml) - Final volume (ml) x 100/initial volume (ml). Technical efficacy (TE) (4) was defined as a VRR ≥50% at 6 months (intermediate follow-up). In the early days of TA of TN, we considered a therapeutic success in case of a reduction in volume ≥50% at one year. With the multiplication of experiments and publications, the target has clearly increased and in our review, the VRR in all of the studies (mainly from Asian and European population) is between 67% and 93.6%. If we position the cutoff at 75%, 11 studies are above and 6 are below. In the main series (≥100 patients), we observed a VVR of 84.1 ± 14.93% (1 to 41 months of FU) (28), 79.4 ± 2.5% (24 months of FU) (23), 93.6 ± 9.7% (49.4 ± 13.6 months of FU) (16), 80.3 ± 13.7% (12 months of FU) (13), 72.3 to 98.7% (16.3 ± 5.6 months of FU) (22), 75% (64.6 – 83.3) (18 months of FU) (27), 69.8% (35 (24–60) months of FU) (33) and 77.1% (60 months of FU) (17). Notice that Bernardi et al. (17) proposes to perform another FNAC for the nodules that failed the treatment. However, TE was achieved in 85% of patients and regrowth occurred in 20% of patients in their study.

The second endpoint of effectiveness is the percentage of volume regrowth after treatment. The terms used are “regrowth”, “marginal regrowth”, “partial regrowth”, “recurrence” (4). A nodule is considered to be recurrent if there is a volume recovery of 50% from the observed nadir. Other authors speak of regrowth when the volume exceeds the procedural volume. This relatively recent data was very little developed in the previous studies. A recent review by Sim (24) have summarized the data and have shown that for 54 patients who underwent RFA treatment, the mean FU period was 39.4 ± 21.7 months, the vital volume increases occurred in 31 nodules (57.4%) and there was regrowth in 13 nodules (24.1%). Cervelli (5) introduces the notion of “residual vital tissue” which, when it is too large, will establish the indication of a second early treatment. Future studies should integrate these data. In our review, the proportion of regrowth ranges from 0 to 34%.

The third endpoint of assessment is the re-treatment of the nodule. In most of the studies, it is not possible to know whether these retreatments are due to a failure of the first treatment or to tissue regrowth, nor the objective criteria which prompted the proposal of a new therapeutic sequence. In Hong’s study (30) given the bilateral nature of the lesions, two procedures were required. He reports that some large nodules were treated in several stages. Deandrea’s study shows a re-treatment rate of 13.6% (RFA or surgery) with a re-growth rate of only 4.1% (33). Bernardi et al. in his recent publication (17) reported a re-treatment rate of 12% with surgery (3.9% non-benign lesion).

The fourth endpoint relates to the quality of life (QOL) indices. Data from the literature indicate that at 12 months, a very significant improvement in QOL and cosmetic score is noted in all studies. Beyond 12 months, a few publications report the maintenance of this improvement or even an improvement in the QOL.

The last endpoint is the evolution of thyroid function in patients treated for a pretoxic or toxic nodule. In the Spieza study (23), 28 patients presented with hyperthyroidism treated with anti-thyroid drugs. Twenty-two recovered their thyroid function without treatment, the other six required a lower dose of anti-thyroid drugs compared to the pre-TAT period. In the Sung study (29), all the patients became euthyroid after treatment, but 8/44 had a relapse of hyperthyroidism despite multiple treatments (up to six for one of them). In Ben Hamou study (27), 10/13 patients treated with RFA for hyperthyroidism became euthyroid. Finally, the toxic nodule in Aldea’s study (34) reacted well initially but fell again and had to be treated with RAI. In a recent series, Dobnig (36) proposed RFA for the treatment of autonomous and toxic nodules under certain conditions (volume in particular). The treatment of pretoxic or toxic nodule has finally be validated by the European Thyroid Association (3), and notably for pregnant women.

### Safety

In all the previous studies, early adverse events are mainly well-described. In Ben Hamou (27), five (3%) major adverse events (two transient recurrent laryngeal nerve palsy, one compressive hematoma and one sub-cutaneous abscess), 25 (15.1%) minor adverse events (seven transient dysphonia, two nodule ruptures with conservative treatment and sixteen benign hematomas), and 101 side effects (60.8%) occurred during the early FU (1 month). Cervelli (5) have shown two transient minor injury of the laryngeal nerve complete recovery after 2 weeks of corticosteroid therapy). In a recent retrospective study (37), one transient vocal cord palsy, one nodular rupture (which was detected one month after RFA as a red cervical lump with a fluid collection), and one vaso-vagal reaction forcing to interrupt the procedure, were observed. In the majority of the studies, early major adverse events occurred in less than 1% to 3% of cases.

The occurrence of hypothyroidism or hyperthyroidism during FU (>18 months of FU) has never been reported in any previous studies.

None of the publications has reported any complications occurring after 12 months of FU. In the article of Aldea Martinez (34), one patient retained the recurrent paralysis that was noticed earlier after the intervention (direct approach, not trans-isthmic).

### Predictive Factors

Valcavi (26) shows that the predictive factor of success is the total energy deposited correlated with the initial volume (negative correlation). On the other hand, no relationship was found with age, sex and volume. In the very recent Italian multicenter study (17), a correlation was found between insufficient results and regrowth, and the low amount of energy deposited [low-energy delivery (optimal cutoff was 918 J/ml for RFA)], young age, large volumes, low VRR at one year. Jung et al. (13) have found that the solidity of the nodule and the energy delivered were independent predictive factors of the final volume reduction. However, many studies lack the information regarding the energy delivered. Very few data concerning the learning curve were mentioned. However, some studies report a higher success rate when the RFA procedure is performed by an experienced physician, with a suggested cutoff of 50 cases. Two recent studies (37, 38) have demonstrated that only baseline volume, total energy, and energy per volume were independently associated to a VRR >50% (*p* = 0.001, *p* = 0.0178, *p*<0.001, respectively) and that delivering 756 and 2670 J/ml gave a probability of VRR >50% in 50% and 99% of patients, respectively. Besides, Russ et al. evokes possible explicative factors: energy applied per volume was positively correlated to VRR and ablation ratio (AR) but not to TE. Ablation duration was associated with a greater reduction of AR but there was no significant correlation with TE and VRR. Lastly, time trend correlation showed a significant increase in delivered energy per volume and ablation duration with experience.

### The Follow-Up

The lower limit was set in this review of the literature at 18 months. Intermediate data are most often taken at 3, 6, and 12 months. In most of the studies, 5% to 15% of the patients are lost to FU. The most important FU was 8 years (Ha) (31): the authors have decided to FU their patients annually [FU period of more than 5 years (mean, 5.8 years; range, 38–98 months)] and have shown a volume reduction rate of 81.3% ± 5.8% (*p<*0.0001).

US was performed, as long as the clinical examination (clinical signs and symptoms) to evaluate late complications (*i.e.*, complications appearing after one month following the treatment). The 2020 ETA guidelines have recommended in recommendation 8: “early-term (*e.g*., 3 months) and intermediate-term (*e.g*., 6 and 12 months) clinical, biochemical, and US evaluations; long-term FU monitoring is suggested, in the absence of symptoms every 1 to 2 years, in order to reveal regrowth,” but finally, physicians do not know clearly how long the patients should be followed up (probably more than 2 years).

## Discussion

### Efficacy and Safety

**Figure 3:**
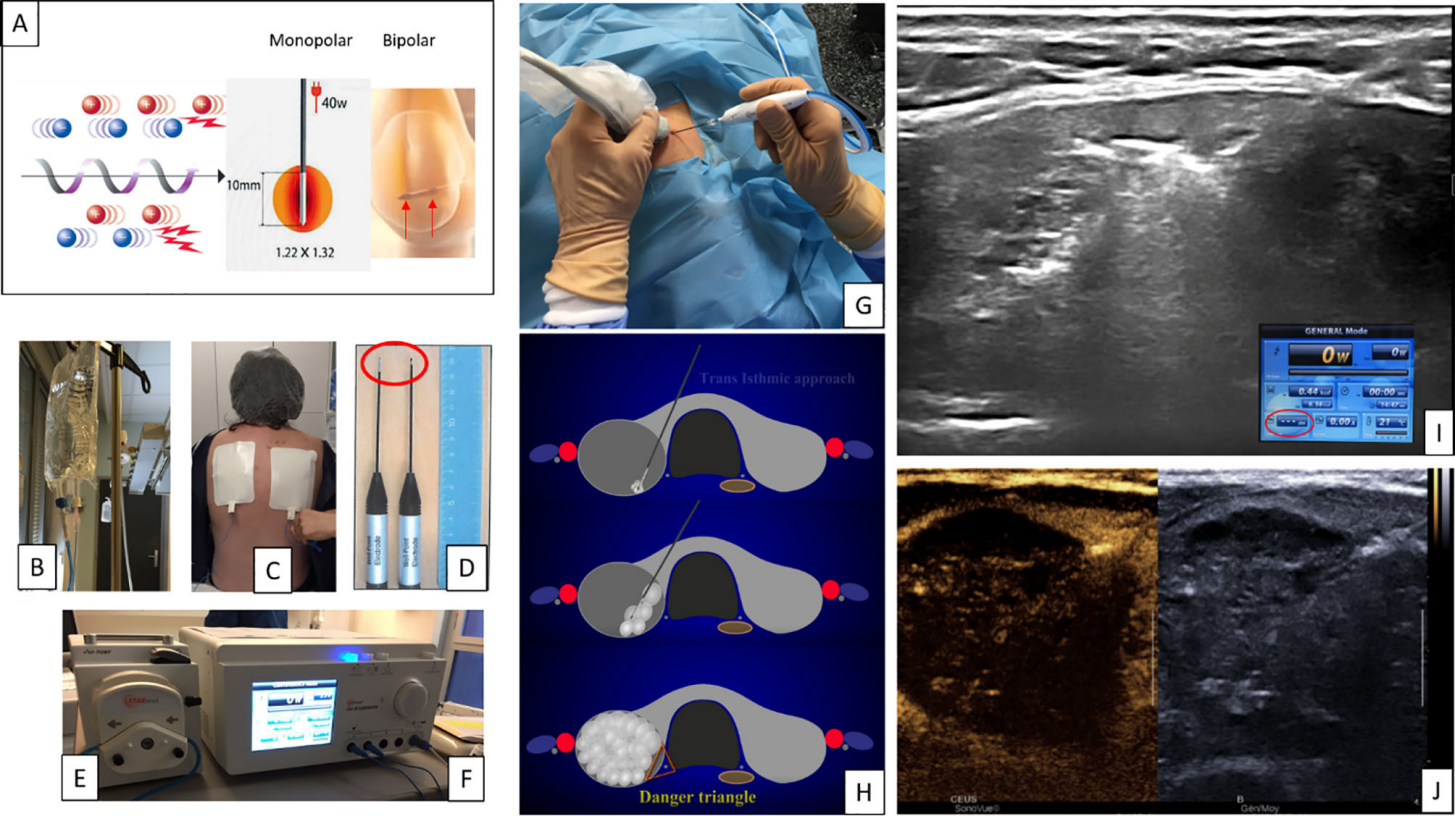
Radiofrequency ablation (RFA): physiology, equipment and ablation. Ionic agitation **(A)** and formation of frictional heat. Monopolar: Using a 40W RF power, and an active-tip of 10 mm, the diameter of the action zone is 13 mm. Bipolar RFA electrode with two tips **(D)**. Internally cooled electrode (active-tip 5 and 10 mm) receives cold fluid **(B)** perfusing by a peristaltic pump **(E)**. The generator **(F)** supplies RF power through the active-tip of the electrode. Grounding pads **(C)** act to disperse electricity in the RF circuit. Using ultrasound guidance **(G)**, the electrode is inserted into the nodule. A trans-isthmic approach **(H)** and a moving shot technique are recommended. A cloudy effect **(I)** and an increase of the impedance (red circle) indicate the moment to change ablation zone. CEUS confirms **(J)** the complete treatment of the nodule (“vascular desert”).

Radiofrequency ablation is now considered as an effective technique, safe, reproducible from one team to another, as an alternative to surgery as part of the management of benign TN, whether solid, mixed (solid/cystic), functional or non-functional (**Figure 3**). Large prospective and retrospective series carried out by Asian (20), European (3) and American (39) teams have demonstrated a significant volumetric reduction of treated thyroid nodules of 60 to 85% or even beyond 85% depending on the series, associated with a significant reduction in the symptomatology attributable to the thyroid nodule (compressive symptoms, cosmetics). RFA is widely used (more than LTA, microwaves and HIFU) probably because of its greater simplicity and reproducibility. However, treatment by thermal-ablation must be carried out within an expert center and requires indisputable ultrasound and anatomical expertise. This minimally invasive technique justifies an expert clinical, biological and ultrasound evaluation before its realization. In fact, several series have highlighted numerous factors predicting success such as the initial size of the nodule, its vascularization, the percentage of residual viable zone (40, 41). It should not be put in competition with surgery but should be presented as an additional therapeutic option which could be positioned as a first-line option depending on *(i)* the nodular characteristics and its clinical application, *(ii)* the patient’s decision. In the current economic context, and to the extent that surgical treatment appears more expensive, it appears justified to propose an alternative which will make it possible to limit the economic impact of the treatment of nodules (which represents the most frequent endocrinopathy). The cost of the HIFU device is around 250*,*000 euros (€) *vs.* 30*,*000 € for LTA, 17*,*000 to 25*,*000 € for RFA and 20*,*000 to 25*,*000 € for MWA; and the cost of disposables is about 500 € *vs.* one fiber 300 to 500 € for LTA, one electrode 700 to 900 € for RFA and one antenna 900 to 1*,*000 € for MWA. But, also to limit the impact of surgery on the patient with all the risks that this implies (hemorrhage, recurrent paralysis, secondary hypocalcaemia, compressive hematoma) (11, 42) and to preserve thyroid function (20% of patients benefiting from lobectomy require hormone replacement therapy and the vast majority of patients undergoing thyroidectomy require lifelong hormone replacement therapy (43). In the majority of studies, a serious adverse event rate of <3% is reported (this prevalence tends to decrease with the experience of the operators). Treatment with TA involves regular clinical and ultrasound monitoring in order to assess the long-term nodular development (VVR) and whether or not it requires a new session. Finally, there are very few series in the literature for which long-term FU (beyond 18 months) is available. It is accepted in its series the need to reprocess the nodules, sometimes twice, three times or even more than three times (**Table 1**). It is therefore advisable, before the procedure, to inform the patient of the possibility of nodular regrowth with the possibility of new TA sessions.

### Optimization of Treatment Methods

Since the first rat trials for the treatment of hyperthyroidism (44) and the first representative series by Jeong et al. (28), techniques and technology have not stopped to improve in order to optimize the management of thyroid nodules. If we go back to the genesis, it is true that in Asia the preservation of the axis of fertility “head-genitals” motivated the development of alternative techniques such as TA but also surgical techniques with alternative approach (in particular axillary) robot assisted (45). From now on, TA is widespread throughout the world and carried out in reference centers which benefit from an accreditation by the Korean learned societies, pioneers in TA techniques. The suppliers and generator developer are constantly improving, with the help of the physicians, the technical methods. There are currently retractable radiofrequency electrodes making it possible to optimize the treatment of the treated zone; in particular in posterior zones and zones that are very close to the capsule. Lee et al. (46) have shown that an adjustable electrode for RFA of benign TN was effective and safe. This will most likely limit the viable residual area after treatment; therefore limiting nodular regrowth (47). Moreover, recent Chinese series have shown a reduction in procedural time when radiofrequency was coupled with prior ethanol injection, with a mean treatment time of 441.30 ± 243.31 seconds *vs.* 790.70 ± 349.82 seconds; t=4.403, *p=0.000*; that prior ethanol injection is effective in reducing nodule volume, relieving compressive symptoms, and cosmetic discomfort (48). Randomized trials will be needed to validate this new combined procedure. Procedures combined with the administration of iodine 131 (in particular in the context of solid AFTN) have already demonstrated their effectiveness (49), extend to develop in particular for large AFTN. Moreover, the achievement of about fifty RFA is necessary to obtain good clinical and ultrasound results (significant reduction of the initial symptomatology and significant reduction of the nodular volume) corresponding to the learning curve (27). It is notably correlated to the power used and to the energy deposited on the nodule which are associated with the appraisal of the operator training.

### Comparison With Other Techniques

Regarding the procedure itself, whether for RFA or LTA, it takes place in a mini-intervention room or in the operating room in the presence or not of an anesthesiologist, more or less with sedation, the patient placed in dorsal decubitus, neck in hyper extension, under ultrasound guidance in aseptic conditions. The approach is trans-isthmic with the radiofrequency electrode with the possibility of using the ‘moving-shot’ technique, for the laser, the laser fiber (s) is (are) positioned using a fixator/trocar. Several studies have focused on the long-term FU of patients treated with laser. The efficiency of the laser in terms of volume reduction is comparable to that of the RFA (50–52). Several comparative studies have been carried out between LTA and RFA over a period of less than 2 years. Other series with a longer FU [18 months for Ben Hamou et al. (27) and 60 months for Bernardi et al. (17)] did not show a difference between the two techniques (VVR between 60 and 85%). However, the risk of nodular regrowth is lower in patients treated with RFA than in those treated with laser. Few studies have focused on percutaneous microwave ablation (PMWA), in the treatment of benign TN. Liu et al. (53) has shown that PMWA is an effective and safe technique in 474 benign solid TN with a mean 90% decrease in volume at 12 months after the treatment. Yue et al. (54) have also concluded the efficacy of PMWA with a signification volume reduction at 12 months after treatment (12.6 ± 15.1 to 3.2 ± 5.7 ml). A single-center retrospective study comparing the efficacy and safety of RFA (40 patients), PMWA (40 patients) and HIFU (14 patients), has shown a slight superiority of RFA, with a mean volume reduction of nodules of 50% *vs.* 44% for MWA and 48% for HIFU at three months after treatment (short period of time). Finally, in a recent series, the authors compared RFA and MWA (53). The RFA group achieved higher VRR than MWA group at 6 and 12 months (77.9 ± 21.0% *vs.* 68.7 ± 19.1% (*p=0.038*), and 85.4 ± 18.9% *vs.* 75.8 ± 19.4% (*p=0.029*), respectively).

### Future Perspectives

The optimization of care and technical innovation is a fundamental point in the management of these patients. Benign thyroid nodules do not represent *a priori* any particular threat for the patient but when they become compressive and/or they increase in size (15% of the nodules progress in volume during the FU), then it is necessary that the patient can benefit from the best therapeutic option. A recent survey (55) by the members of the European Thyroid Association suggested that TA was the preferred option only for a 4-cm benign nodule in old patients with comorbidities. The objective is therefore to improve the diffusion and the access to these new minimally invasive techniques. Recent studies concerning patient satisfaction (QOL questionnaire) after treatment have all mainly shown patient satisfaction (22) concerning the technique. It has also been recently shown that there is no risk of cancer after treatment of a benign thyroid nodule by TA. In fact, Ha et al. (31) showed that at five years after RFA, no atypical cells nor neoplastic transformation were detected in the undertreated peripheral portion of treated benign nodules on the CNB specimen, concerning 16 nodules. On histo-pathological examinations, 81.2% of acellular hyalinization was observed.

The indications for thyroid RFA are now broadening with the treatment of papillary thyroid micro-cancers (PTMC) and the treatment of cervical lymph node recurrence. Indeed, Cho et al. (56) have shown the efficacy and safety of RFA in the management of low-risk papillary cancers (84 PTMC from 74 patients) with complete disappearance rates of 98.8% and 100% achieved at 24 and 60 months respectively after the treatment. No occurrence of local tumor progression, lymph node, or distant metastasis were reported, and no delayed complications, procedure-related death, or delayed surgery occurred over the 72-month mean FU period. In patients with PTMC (57), RFA appears to have an advantage over surgery in terms of QOL, supporting the role of RFA as an alternative strategy to surgery. Another study (58) showed the efficacy and safety of RFA in the treatment for low-risk PTMC after a long-term FU period (42.1 ± 11.8 months); with a mean VRR of 98.8 ± 6.4%. After RFA 366 tumors (88.4%) completely disappeared. One patient had a residual cancer, four have developed metastatic lymph nodes, and ten patients had recurrent PTMC. Thirteen of those patient were retreated by RFA, with a complete disappearance of 11 lesions during FU. Moreover, RFA is considered as an effective and safe minimally invasive therapy of locally recurrent papillary thyroid cancer since a mean volume reduction of 99.5% ± 2.9% was observed and that 46 treated tumors (91.3%) had completely disappeared by the final evaluation (59). Lim HK et al. have shown similar results suggesting the efficacy of RFA treating low-risk PTMC in patients who are of high surgical risk or refuse surgery. They finally observed a complete disappearance of ablated tumors found in 91.4% (139/152) (60).

## Conclusion

Thermal ablation is a safe and effective method for the treatment of benign thyroid nodules and radiofrequency seems to be one of most efficient techniques. The volume reduction rate can reach 80% of the initial nodule volume in most studies but the patient should be informed that a re-treatment may be needed in case of huge nodules or incomplete ablation. Minor adverse events are frequent but usually reversible while major complications are rare (<3% of cases). So, these findings represent a clear advantage versus the incompressible rate of surgical complications and the duration of sick-leave (about 10 to 21 days for surgery).

All the studies under examination consistently validated the long-term clinical efficacy and the substantial safety of RFA for the treatment of benign thyroid nodules. Thermal ablation, however, is an operator-dependent technique and should be performed in centers with specific expertise. The selection of patients should be rigorous because the nodule size and the structural and functional characteristics influence the appropriateness and the outcomes of the treatment. Future perspectives as the treatment of micro-papillary thyroid cancer or cervical recurrence need further investigations.

## Data Availability Statement

The raw data supporting the conclusions of this article will be made available by the authors, without undue reservation.

## Author Contributions

HM and ABH wrote the first version of the manuscript. ABH, HM, and AA contributed to the review of the literature. All authors contributed to the article and approved the submitted version.

## Conflict of Interest

HM has previously consulted for THERACLION and STARMED (oral conference communication).

The remaining authors declare that the research was conducted in the absence of any commercial or financial relationships that could be construed as a potential conflict of interest.
